# Current Strategies to Control Recurrent and Residual Caries with Resin Composite Restorations: Operator- and Material-Related Factors

**DOI:** 10.3390/jcm11216591

**Published:** 2022-11-07

**Authors:** Moataz Elgezawi, Rasha Haridy, Moamen A. Abdalla, Katrin Heck, Miriam Draenert, Dalia Kaisarly

**Affiliations:** 1 Department of Restorative Dental Sciences, College of Dentistry, Imam Abdulrahman Bin Faisal University, Dammam 31441, Saudi Arabia; 2 Department of Clinical Dental Sciences, Princess Nourah Bint Abdulrahman University, Riyadh 11671, Saudi Arabia; 3 Department of Conservative Dentistry, Faculty of Dentistry, Cairo University, Cairo 4240310, Egypt; 4 Department of Substitutive Dental Sciences, College of Dentistry, Imam Abdulrahman Bin Faisal University, Dammam 31441, Saudi Arabia; 5 Department of Conservative Dentistry and Periodontology, University Hospital, LMU Munich, Goethe Str. 70, 80336 Munich, Germany

**Keywords:** recurrent caries, residual caries, caries management, resin composite, materials technology, biodegradation resistance, biomimetics

## Abstract

This review addresses the rationale of recurrent and/or residual caries associated with resin composite restorations alongside current strategies and evidence-based recommendations to arrest residual caries and restrain recurrent caries. The PubMed and MEDLINE databases were searched for composite-associated recurrent/residual caries focusing on predisposing factors related to materials and operator’s skills; patient-related factors were out of scope. Recurrent caries and fractures are the main reasons for the failure of resin composites. Recurrent and residual caries are evaluated differently with no exact distinguishment, especially for wall lesions. Recurrent caries correlates to patient factors, the operator’s skills of cavity preparation, and material selection and insertion. Material-related factors are significant. Strong evidence validates the minimally invasive management of deep caries, with concerns regarding residual infected dentin. Promising technologies promote resin composites with antibacterial and remineralizing potentials. Insertion techniques influence adaptation, marginal seal, and proximal contact tightness. A reliable diagnostic method for recurrent or residual caries is urgently required. Ongoing endeavors cannot eliminate recurrent caries or precisely validate residual caries. The operator’s responsibility to precisely diagnose original caries and remaining tooth structure, consider oral environmental conditions, accurately prepare cavities, and select and apply restorative materials are integral aspects. Recurrent caries around composites requires a triad of attention where the operator’s skills are cornerstones.

## 1. Introduction

Caries is the most widely prevailing noncommunicable disease [1], and is a multifactorial process presently considered as biofilm-mediated rather than an infectious disease [2,3]. According to the ecological plaque hypothesis, caries occurs as a result of an imbalance of oral microflora—normally more than 700 species—leading to an increase in cariogenic bacteria such as *Streptococcus mutans* and *Lactobacilli* types [4]. The accumulating cariogenic bacteria produce acids such as lactic acid that reduce the local pH, leading first to demineralization and later to the destruction of the organic matrix. Caries progresses when demineralization cycles prevail and remineralization cycles cease [5]. Classically, bacterial proteases are blamed for the proteolytic process taking place because of dental caries. Recently, it has become increasingly evident that activated endogenous matrix metalloproteinases (MMPs) and cystine cathepsins of salivary, gingival crevicular fluid and dentinal origin, together with bacterial proteases, share in degrading the dentin matrix of demineralized dentin. This takes place at neutralized pH levels where the buffering effect of saliva takes place, since MMPs operate only in neutral pH values [6,7].

Traditionally, caries was considered a progressive process, necessitating its complete removal during cavity preparation. The modern understanding of caries as a preventable and reversible disease at the initial non-cavitated phase has directed attention towards preventing its incidence and reversing initial enamel lesions via ion precipitation remineralization. Moreover, the possibility to remineralize partially demineralized dentin collagen in caries-affected dentin made it possible to adopt a more conservative selective caries removal strategy. Conservative approaches such as stepwise and partial caries removal aiming to preserve the vitality of the pulp in deep carious lesions are more extensively employed. More recently, modern biomimetic remineralization approaches have achieved success in remineralizing completely demineralized dentin matrix [8,9].

While primary caries describes carious lesions occurring on intact tooth surfaces, secondary or recurrent caries refers to lesions developing in the tooth structure adjacent to an existing restoration, either as surface lesions at the margin or in close vicinity to the restoration, or internally as a wall lesion at the tooth–restoration interface [10]. Whether recurrent caries is marginal or in walls at deeper locations, there is a lack of precise identification of the marginal gap size or nature of the internal wall defect or surface effects predisposing recurrent caries [11,12]. Although the clinical discrimination between recurrent and residual caries is impractical, a clear differentiation between the two terms has been preferred and considered in some reports and review articles [13,14].

Resin composites are currently the most widely used restorative materials due to their adequate mechanical properties, their satisfactory esthetic qualities and improved adhesive resin material technology. However, polymerization shrinkage, technique sensitivity and the progressive biodegradation of resin composites and the deterioration of resin bonding to the tooth structure are continuing challenges that eventually lead to failure of the restoration. The need for new resin composite materials and clinical strategies that consider the existing oral environmental challenges remains an interest of researchers and clinicians. The aim is to assure long-term clinical reliability and patient satisfaction, and to minimize the risk of failure [15].

Recurrent caries is known to be a main form of failure and justification for the replacement of resin composite restorations, together with restoration fractures. Recurrent caries around resin composites is one of the adverse consequences of microleakage. The likelihood of recurrent caries appears to be correlated to the marginal and interfacial gap size, as well as to the mechanical loading during functional mastication. Recurrent caries is a complex multifactorial process that requires a thorough analysis of the tooth and the restorative material, as well as the chemical and bacterial effects of the oral environment [16]. The FDI commission 2-95 has outlined three factors influencing the quality of dental restorations: patient-related, operator-related and material-related factors [17]. Although reports have highlighted the role of patient-related factors in the incidence of recurrent caries around resin composite, material-related factors are critical and can result in different scenarios of incidence of recurrent caries [13,18].

The purpose of this study was to display and discuss the material- and operator-related factors that influence recurrent caries incidence and the fate of residual caries with resin composite restorations.

## 2. Methodology

PubMed and MEDLINE databases were searched for composite-associated recurrent/residual caries. The focus was placed on predisposing factors related to materials and operator’s skills; patient-related factors were out of scope. Systematic reviews, clinical trials, observational studies and relevant in vitro studies were targeted. The following keywords were employed in the search: recurrent caries, residual caries, caries management, resin composite, materials technology, biodegradation resistance, biomimetics. This article is a narrative review; therefore, the included literature was related to the keywords and only patient-related factors were excluded. Strict inclusion and exclusion criteria such as those applied in systematic reviews were omitted. Most of the included literature was published after the year 2000.

This article critically reviews and evaluates the information available from existing studies of recurrent caries related to resin composite restorations. Relevance is made to evidence-based recommendations of primary deep caries management, the fate of residual caries, resin composite material-related factors, and contemporary developments. In addition, various techniques for inserting resin composites to improve material performance and control recurrent caries are evaluated. Patient-related factors are beyond the scope of this narrative review. A distinction between residual remaining caries and recurrent caries will follow in respective sections.

## 3. Minimal Invasive Management of Deep Caries and the Fate of Residual Caries

The process of cavity preparation has a deciding influence on the quality of outcome and the longevity of composite resin restorations. Proper cavity design and suitably completed preparation require thorough consideration of the different biomechanical and esthetic aspects of the tooth in concern, as well as the influencing factors of the oral environment. Nevertheless, full recognition of the extent of the carious lesion and the amount of remaining sound tooth structure is crucial. In this regard, effective rubber dam isolation before cavity preparation is mandatory whenever possible to assure adequate visibility, sound judgement and accurate fulfillment. An appropriately prepared cavity is a prerequisite for optimum bonding, good adaptation, and an effective marginal seal [19].

The European Organization of Caries Research (ORCA) and the International Association of Dental Research (IADR) have recently discussed and agreed on the most appropriate definitions related to caries based on the present concepts and modern understanding of dental caries and associated managements [20]. Accordingly, primary caries is a carious lesion in a previously sound tooth surface, while secondary or recurrent caries is a carious lesion that has developed adjacent to a restoration, and residual caries is a demineralized carious tissue left in place before the restoration is placed [19].

Secondary or recurrent caries are two interchangeably used terms describing carious lesions that are principally divided into two categories: surface and wall lesions. The old concept of complete or non-selective caries removal to hard dentin requires the excavation of caries to hard dentin in the entire cavity [20]. The removal of all carious tissues and extending cavity margins to presumably less caries-prone areas of the tooth surface to prevent caries recurrence is currently considered an unjustifiable and radical approach that sacrifices the biomechanical and esthetic integrity of the tooth structure. Conversely, more conservative approaches are adopted that minimize surface extensions to a minimal intervention approach, while at the same time limiting caries excavation to infected dentin, leaving behind the affected remineralizable dentin [20].

### 3.1. Partial and Stepwise Caries Removal

This paradigm shift in dental caries management has been widely accepted to replace the old concept of “extension for prevention”. A current scale of recommendations and options exists to treat extensive carious lesions, limiting removal to heavily infected and necrotic dentin and maintaining the remineralizable caries-affected dentin. The objective is to preserve the vitality of the pulp and extend the life span of the tooth as a functioning unit in the dental arch [19,20]. Strong evidence of systematic reviews, meta-analyses and clinical trial studies have demonstrated the high success rate of selective or partial caries removal procedures over complete caries removal. Cumulating evidence continues to support incomplete caries removal and discourage complete caries removal in deep cavities. Regardless of the controversy over whether or not residual infected caries is arrested in cavities sealed with restorations, it is more important to create effective and reliable restorations than to completely remove caries [21]. In a recent clinical study, it was found that the bacterial load under restorations was initially lower with complete versus selective caries removal, but was similar in both cases three months after the cavities were sealed with restorations [22].

Modern conservative deep caries treatment comprises two techniques: partial caries removal and stepwise caries removal. Partial caries removal is a procedure by which dentin caries is removed from the outer zones of a deep cavitated caries lesion (excavated to hard dentin), followed by the partial removal of soft dentin from the pulpal wall with a hand excavator or round bur. Treatment is indicated for deep dentin lesions to avoid pulp exposure [19]. In partial caries removal there is no second visit, and initial caries removal is followed by sealing the restoration with a final restoration [23].

Stepwise caries removal, on the other hand, is caries excavation in two (or more) steps, with a time interval between the steps to stimulate mineral deposition in the dentin prior to final excavation. In the first visit, the surrounding cavity walls are excavated until hard dentin is reached, while only the necrotic, disorganized dentin is removed at sites of proximity to the pulp until the level of soft dentin is reached. The cavity is then sealed with a provisional restoration over a period of 6 weeks to 12 months. The first step is partial caries excavation followed by additional caries removal to a firm or leathery dentin in the later visit, before insertion of the final restoration [20]. The literature shows some evidence that the success rate of one-visit partial caries removal is higher than stepwise caries excavation [24,25].

Although partial caries removal involves leaving a layer of infected dentin for the sake of preserving the vitality of the pulp in deep caries with a high risk of pulp exposure, cariogenic bacteria beneath clinically reliable restorations will eventually die or become markedly inactive [24].

The clinical identification of caries zones, where texture correlates with the degree of infection and the viability of dentin tissue, remains a crucial guideline for conservative caries management. Soft caries is similar to cottage cheese in texture, readily deforms upon pressing with a hand instrument, and can be easily peeled off with excavators indicating infected dentin with dissembled collagen framework cross-links [26]. Leathery dentin does not yield upon pressing and needs higher pressure to be removed with an excavator and lays in the middle of the range between soft and firm dentin. On the other hand, firm dentin is more resistant to physical pressure and needs more pressure for lifting by hand excavators. Leathery/firm dentin indicates caries-affected dentin with sound remineralizable collagen plexus configurations [26]. Hard dentin is a sound healthy dentin that requires a sharp cutting edge or a bur to be removed and has a scratchy sound of “cri dentaire” upon probing [27].

### 3.2. Clinical Endpoint of Dentin Caries Excavation

A main challenge of partial and stepwise caries removal procedures is the clinical endpoint of dentin caries excavation that frequently relies on the subjective tactile sensation of the soft, leathery, firm, and hard dentin with a lack of exact correlation with the actual histopathological features of dentin caries [2,20,28,29].

In a survey performed in three European countries in 2016, most dentists found dentin color inadequate as a criterion of caries removal, reporting that they rely on dentin hardness to assess dentin caries excavation with an aim of reaching hard dentin in proximity to pulp [30]. On the other hand, a survey conducted in 2015 found a wide range of variety between dental schools’ programs regarding the management of deep caries and the exact definition of caries remaining at deep cavity sites. The study indicated the need to establish consistency between cumulating evidence and teaching, as well as the calibration of examiners upon evaluating cavities with deep caries [31].

**Figure 1:**
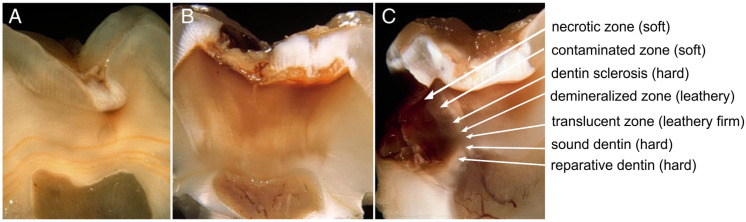
Progress of caries (**A**,**B**); reactions and different zones of dentin caries (**C**); reprinted with permission from Bjørndal et al. published by Elsevier 2014 [29].

Evidence-based reports recommend two possible clinical endpoints of dentin caries excavation in partial caries removal procedures. The first one is selective caries removal to soft dentin, which is indicated in deep caries that has progressed to the inner third of dentin with a high risk of pulp exposure upon complete caries removal. In this case, a limited layer of infected soft dentin is left behind near the pulp to avoid pulp exposure and maintain pulp vitality. The second is selective caries removal to leathery/firm dentin (physically resistant to hand excavation). This is indicated in moderate-depth lesions that have not reached the inner third of the dentin. In all cases, however, the surrounding walls of the cavity should be excavated to hard sound dentin [32], Figure 1.

Using liners of calcium hydroxide or resin-modified glass ionomer after partial caries removal in deep carious lesions may not be an essential prerequisite for the clinical success of the procedure [33]. Conversely, calcium hydroxide and glass ionomer liners proved successful in reducing the number of viable bacteria remaining and the quality of residual caries left behind after partial caries removal [34]. To arrest residual caries and enhance remineralization in deep carious lesions managed by partial caries removal, ozone and silver diamine fluoride treatments were suggested [35,36,37]. Further studies are needed to confirm procedural effectiveness, any adverse effects on bonding, biocompatibility, or deep penetration, and esthetic consequences [38].

A liner of glass ionomer can impart a required antibacterial potential because of the fluoride content. On the other hand, the use of cavity cleansers in cases of partial caries removal procedures can provide antibacterial and antiproteolytic activities, thus improving resin–dentin bond stability and reducing the number of viable bacteria remaining in dentin. On the other hand, materials such as MTA and calciumhydroxyde are effective as pulp-capping materials for their therapeutic effect in inducing reparative dentin formation. Therefore, it is recommended to use them when direct or indirect pulp capping is needed [39,40,41,42].

The rationale in conservative deep caries management is to achieve an intelligent balance between the protection of pulp vitality and the avoidance of pulp exposure due to overzealous caries excavation from one side and attaining reliable bonding and a tight seal by excavating an adequate amount of carious dentin on the other side. A proper diagnosis of the pre-existing status of the pulp, adequate history taking, pulp sensibility testing, a percussion test, and radiographic examination are essential to confirm normal vital pulp before deep caries management procedures take place [43]. For the successful minimally invasive management of deep carious lesions, effective marginal seal and reliable bonding must be achieved.

Based on the new understanding of caries as biofilm-induced rather than an infectious disease, residual or remaining dentin caries beneath an existing restoration are not considered a failure since a peripheral well-sealed restoration is more clinically relevant [2,13,44]. In this regard, the presence of radiolucent zones beneath composite restorations are not considered a justification for restoration replacement [45].

Systematic reviews, meta-analyses and clinical trials reveal that evaluating partial and stepwise caries removal are based on collecting symptoms of pain and the assessment of clinical signs of adverse pulp reactions. Clinical procedures of inspecting the presence of swelling or fistulous tract, pulp testing, palpation and percussion tests, in addition to radiographic examination, are usually employed. However, there is inadequate evidence regarding the risk of the future failure of restorations with incomplete and stepwise caries removal [46]. Moreover, there is a lack of exact consistency between subjective clinical signs and symptoms and the actual histopathological status of the pulp tissue. Pulp reaction to dental caries starts as early as enamel caries and advances as caries progresses. Therefore, a careful clinical evaluation of pulp status is mandatory in deep caries evaluation [3,29,47] (see Figure 1).

Well-designed long-term clinical studies are needed to validate the future risk of restoration failure and the development of irreversible adverse pulp consequences following these procedures [48]. This is particularly true with high-caries-risk individuals. An exact distinction between new recurrent caries and residual caries beneath an old restoration is missing. The sensitivity and specificity to radiolucent lines beneath a resin composite restoration was less than 80% [45].

Research displayed that bacteria remain in the dentinal tubules after cavity preparation without indicating that the remaining bacteria predispose the progression of caries or restorative failure. The natural defense mechanism of dentin sclerosis in slowly progressing caries and laying down tertiary dentin on the pulpal side of deep lesions helps prevent further bacterial invasion. Nevertheless, the number of bacteria in the superficial zones of caries is much greater than in deeper caries zones, indicating the judicious removal of heavily infected non-viable degenerated dentin [47]. Longitudinal research indicates that the proper isolation of cariogenic bacteria from nutritional resources by an integrated restoration carries no risk of future caries progression. The use of cavity disinfectants such as glutaraldehyde and chlorohexidine is of limited or no benefit if clinically significant marginal gaps and evident microleakage are encountered [24,49].

Several techniques could help in decision-making for the endpoint of caries removal in selective caries excavation [50,51]. Caries detection dyes, polymeric and ceramic burs, and chemomechanical caries removal are among the suggested mechanisms. Fluorescence-aided caries excavation (FACE) uses orange–red fluorescence as a sign of heavy bacterial infection and a barometer for caries removal until the level of green sound dentin fluorescence [3,52,53]. FACE is an effective tool to assess residual caries in vivo [54]. FACE and polymer burs are currently employed by undergraduate students in some dental schools to treat teeth with deep caries. A clinical and microbiological assessment found polymeric burs more efficient in deep caries excavation than a chemomechanical technique [55]. Caries removal with the self-limiting polymer bur does not interfere with effective bonding to dentin [56]. The bonding performance to residual dentin caries varies between caries removal techniques regardless of the reported improvements in bond strength values of different bonding agents. Moreover, there is no completely reliable diagnostic tool for assessing the fate of residual caries [50].

In vitro FACE is more effective in caries removal than a caries detector dye and conventional caries excavation when considering the quantity of remaining bacteria [57]. The use of DIAGNOdent light fluorescence technology to guide caries removal using an Er:YAG laser with a threshold reading of 7 for circum-pulpal dentin left behind dentin collagen with intact links denoting vital dentin [58,59].

Determining an optimum endpoint upon deep caries removal in teeth with normal vital pulp is a focus of interest for researchers and clinicians. It has been recommended to use anatomical and histopathological knowledge together with caries detection dyes and light fluorescence. The objective is to preserve pulp vitality and limit deep caries removal to heavily infected layers. Preventing the progress of residual caries in partial and stepwise caries removal mandates durably effective bonding to dentin and tightly sealed margins with prepared cavities of peripheral hard dentin walls and sound non-carious enamel [60]. The successful management of deep caries should consider that bonding to caries-affected dentin is 33% weaker than bonding to hard dentin due to the adverse resin penetrability of the decreased mineral content of caries-affected dentin. Infected dentin, on the other hand, has a weak disorganized structure and exhibits 78% less bond strength than hard dentin [61].

To determine an objective clinical endpoint during dentin caries removal and to assess the clinical effectiveness of some conservative minimal intervention caries excavation techniques, a microCT study was conducted. The studied techniques included round tungsten carbide bur, tungsten carbide bur with caries detection dye, an air scaler with oscillating tungsten carbide tips, Carisolv chemomechanical caries removal with a mac-tips Carisolv instrument, CeraBur ceramic bur with a self-limiting ability endpoint, a Er:YAG laser, and three suggested experimental methods using hand metallic or plastic excavators. The study found the Er:YAG laser aided by laser-induced fluorescence to be most effective as a selective caries removal technique and that rotary burs with or without caries detection dye are aggressive methods of caries removal [62]. On the other hand, CeraBur and Carisolv were even more conservative, indicating that chemomechanical caries removal is superior to selective caries removal with the preservation of hard sound dentin [62]. Educational programs in different dental colleges around the world still face controversy in teaching and in the clinical assessment of students regarding the management of deep carious lesions. An increasing trend towards the conservative management of deep carious lesions in different dental educational programs is evident [31,56,63].

Papain-based chemomechanical caries removal gel has encouraging potential to effectively and more conservatively remove caries than conventional mechanical caries excavation methods [64]. However, a cell culture study found papain-based gels with some cytotoxicity to dental pulp cells [65]. A recent clinical trial compared two chemomechanical caries removal agents, sodium hypochlorite and a papain-based enzymatic gel Brix 3000 with conventional low-speed burs for caries removal. Both gels perform significantly better than conventional caries removal, with a similar performance [66].

A systematic review and meta-analysis conducted in 2016 found almost one-half of dentists refuse the evidence-based recommendations of selective/incomplete removal of caries. The study recommended progressive investigation with qualitative elements for a deeper understanding of the barriers against the broader implementation of less invasive deep caries management [67].

### 3.3. Diagnosis of Recurrent Caries

The diagnosis of recurrent caries at its early stage is crucial to avoid failed restoration. A clinical review and meta-analysis found that although recurrent caries is an obvious dental health problem, its detection has been investigated by a limited number of studies with little information about the validity and appropriateness for clinical use. The study concluded that visual, radiographic and laser fluorescence might be valuable diagnostic measures of recurrent caries and that the appropriateness of tactile monitoring and quantitative light-induced fluorescence needs further confirmation [68].

In vitro, cone beam computed tomography (CBCT) is effective at detecting secondary caries in occlusal resin composite restorations with less reliability in MOD restorations [69]. Moreover, in MOD resin composite restorations, CBCT is more effective in the detection of recurrent caries than digital radiography [70]. There is a poor-to-moderate agreement between evaluators of CBCT for recurrent caries detection in extracted teeth, although it is more effective than digital radiography [71]. Furthermore, CBCT’s higher radiation dose than digital radiography adds to the impracticality of using it clinically for recurrent caries diagnosis [13]. In vitro, swept-source optimum coherence tomography (OCT) could detect caries beneath composites with a limited depth of imaging and inaccuracy in examining deep restorations [72]. Near-infrared transillumination and reflection at wavelengths from 1300 to 1700 nm showed potential for detecting secondary caries in the first in vitro studies [73]. Presently, there is a shortage of consensus and reliable standards and strategies for the accurate diagnosis of recurrent caries.

## 4. Clinically Challenging Class II Cervical Margins

The literature indicates that caries recurrence is one of the most frequent reasons for the failure of restorations, regardless of the kind of restoration. Recurrent caries is more frequent with resin composites than with amalgam, which suggests that the material contributing factors influence the incidence of recurrent caries, regardless of the patient’s risk status of caries [74]. Studies of recurrent caries around resin composites list several contributing factors, including resin composite type, site, size, and location in the oral cavity in the anterior or posterior teeth, with variabilities in patients and dentists. Several concerns are raised to validate the preciseness of studies of recurrent caries sites. This includes the study period and the nature of the original tooth defect. Some studies report that class V composite restorations show less recurrent caries while most of the restorations are for non-carious cervical defects in otherwise good oral hygiene patients. Nevertheless, it is generally agreed that resin composite restorations in posterior teeth particularly at the cervical margins of class II cavities are the most frequent sites of recurrent caries with resin composites. Optimally restoring the cervical margins of class II resin composites with sustained reliability is a clinical challenge. Among the complicating factors are the development of marginal gaps due to polymerization shrinkage, poor bonding quality because of inadequate light curing at this deep location, and difficulty in establishing a dry field during bonding procedures, especially in subgingivally extending lesions. The absence or inadequate thickness of enamel for more effective bonding is another obstacle to achieving optimal cervical bonding which is aggravated by the degradational influences of the bacterial biofilm, endogenous MMPs, and cysteine cathepsins [2,13,75]. This might explain why the gingival margin of class II resin composite has the greatest vulnerability to recurrent caries [76].

**Figure 2:**
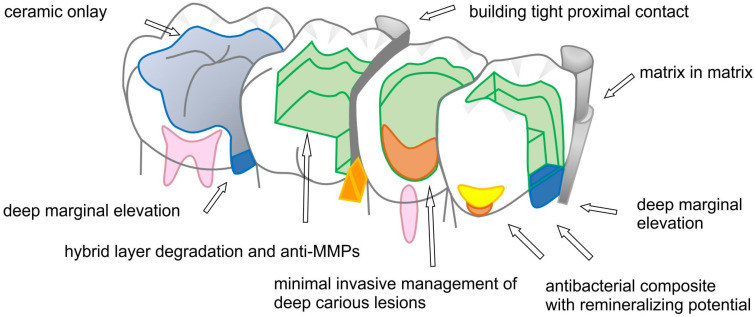
Schematic drawing summarizing most common sites of occurrence of recurrent caries and residual caries.

In clinical practice, the bonding of composite to dentin at the gingival wall is weaker and more friable than in reports of in vitro studies of bond strength values. Bonding to gingival dentin walls is generally less effective than bonding to axial walls and might not be sufficient to resist interfacial polymerization contraction stresses. Adhesive bond strength studies combining in vivo insertion with in vitro testing indicate a greater incidence of debonding at gingival walls of composite resin restorations [11,77,78]. The greatest risk of recurrent caries is at the gingival margins of restorations, irrespective of the kind of restoration. The risk of recurrent caries increases as the gingival margin of the prepared cavity extends more apically from supragingival locations in enamel to deeper locations subgingivally at the cementoenamel junction and cementum. Figure 2 displays a schematic drawing summarizing the most common sites of occurrence of recurrent caries and residual caries.

## 5. Polymerization Shrinkage and Adverse Consequences of Marginal and Internal Gaps

Resin composites are composed of an organic polymeric matrix, inorganic filler particles, and silane coupling agents [79]. As a result of a polymerization reaction, monomers bond into a three-dimensional network of polymeric chains filled with inorganic fillers. The polymerization reaction is accompanied by volumetric shrinkage as the polymer’s chains form. Upon initiation of the polymerization reaction, the C=C double bonds in the dimethacrylate monomer molecules that form the organic phase of most dental composites convert into a C-C single bond with polymer chain formation as additional polymerization reaction progresses [80,81]. During polymerization, when a resin composite is restricted from contraction by the surrounding cavity wall confinements following bonding procedures, interfacial contraction stresses develop at the interface. Marginal and interfacial contraction gaps can arise when these stresses surpass the interfacial bond strength [82].

Interfacial gaps may predispose to recurrent caries in the form of wall lesions. In in vivo study models, caries does not develop in perfect composite–adhesive–dentin bonding, but wall lesions caries occur in all sites of faulty bonding with interfacial gaps [83]. Microleakage due to contraction gaps may also lead to post-restoration hypersensitivity, marginal discoloration, and adverse pulp reactions. Moreover, polymerization contraction stresses may induce pulling action on cusps and the cracking of tooth structure [84]. Not all marginal and interfacial gaps can cause recurrent caries, and there is no general agreement on an exact threshold of gap size for the incidence of recurrent caries [11,85]. However, interfacial gaps larger than 60 μm might lead to interfacial demineralization [13]. In all cases, the patient’s caries risk status is a critical decision-making factor when recurrent caries is considered [85].

The interfacial stresses due to the volumetric polymerization shrinkage of composites against bonded side walls of the cavity are influenced by many factors. These include the type of resin composite, the chemistry of the organic matrix, the technique of insertion, cavity configuration factor (C-factor), the type of adhesive bonding agent, and the nature of the substrate, i.e., tooth surface enamel or dentin. The cavity configuration factor (or C-factor) accounts for the ratio between bonded to free surfaces [86]. Increasing the number of confining constraints of bonded surfaces predisposes the patient to higher polymerization contraction stresses and an increased risk of marginal and interfacial gap development. However, the correlation between C-factor and polymerization contraction stresses should consider the compliance of the prepared tooth structure that varies at different cavity locations [82].

**Figure 3:**
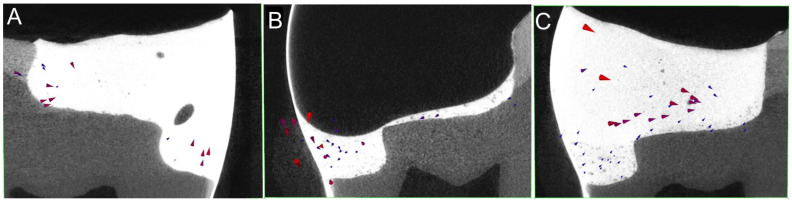
The shrinkage vectors point in the direction of mass movement upon polymerization of resin composites. Bulk application of a bulk-fill composite resulted in an upward movement at the gingival margin of the proximal box (**A**). The application of a thin flowable liner (**B**) below the bulk application of a hybrid bulk-fill composite resulted in smaller shrinkage vectors in the proximal box and yielded a more favorable shrinkage movement toward the tooth–restoration interface (**C**); image reprinted with permission from Kaisarly et al., published by Elsevier 2022 [93].

The non-destructive assessment of resin composite polymerization shrinkage, shrinkage vectors, and interfacial gaps was facilitated using a hybrid technology of microCT scanning and digital image analysis. This opened the door for studying the shrinkage patterns of various resin composites and bonding agents with different cavity boundaries, cavity configurations and material insertion techniques [87,88,89,90]. In large occlusal cavities with undermined enamel, debonding from the cavity floor is observed due to shrinkage away from the cavity floor [91]. Even different composite application methods influence the shrinkage patterns and vector length values. Bulk applications yield larger shrinkage vectors than incremental applications, but material-related factors such as the volumetric shrinkage, shrinkage stresses and time to gelation should be considered [87,92,93,94] (Figure 3).

### 5.1. Protocols to Improve Marginal SEAL and Interfacial Bonding

A biomechanically and esthetically reliable resin composite restoration should be able to restore hard tooth structure defects effectively and durably. Moreover, it should bioactively integrate with the tooth hierarchical complex and surrounding environment, mimicking natural tooth structure construction and physiological biofunction [95,96]. The literature shows several protocols to improve the performance of resin composite restorations and minimize the future risk of recurrent caries. Ongoing research is based on a modern understanding of dental caries, bonding and resin composite material technology, as well as contemporary approaches to biomimetic restorative dentistry and bioactive integration.

In resin composite restorations, an effective and long-term peripheral and internal seal preventing or minimizing marginal and interfacial gaps calls for two basic strategies of clinical protocols. These are maximizing bond effectiveness and reducing the development of interfacial stresses [97]. Resin composites are technique-sensitive materials. Maximizing bonding effectiveness and minimizing interfacial stresses requires meticulous attention to the details of the bonding procedure. Full recognition of the individual oral environmental factors is instrumental. It is the responsibility of the restorative dentist to select the most suitable resin composite material and restoration technique, and choose between etch-and-rinse, self-etch and selective (enamel etching) strategies. The aim is to provide clinically effective tooth–resin composite adhesive junctional complexes with long-term stability [89,98].

Different techniques can improve bonding, reduce interfacial polymerization contraction stresses and improve adaptation at the critical cervical margin of class II resin composites. This includes assuring an adequate degree of conversion of light cure materials. A poor degree of conversion of resin composite restorative materials and bonding agents deteriorates the physicochemical properties of the material, leads to poor bonding, and increases the risk of future caries recurrence [99]. Ideally, the light-curing tip should be close, less than 1 mm away from the surface of the resin composite. The greater this distance, the less light energy that reaches the material to produce an adequate degree of conversion. At greater than 5 mm cavity depth, such as in deeply inserted resin composite increments in deep cavity locations, extra care should be given during light curing. In locations such as the gingival seat of class II cavities or in core build-ups, curing light exposure time should be increased or a dual-cure resin composite might be preferable to assure an adequate degree of conversion [100,101,102].

For adequate curing light energy, an incremental insertion of a maximum 2 mm thick increment of a conventional composite is recommended. Alternatively, bulk-fill composites can be cured as a bulk insertion with a 4–5 mm thick increment. Manufacturers of bulk-fill composites recommend using a light-curing unit with a minimum energy output of 1000 mW/cm^2^ to attain adequate curing at the deep proximal parts of class II cavities for 40 s. Three-directional curing by providing an occlusal light-curing exposure for 40 s followed by a second one from a buccal direction and a third one from a lingual direction can also help to attain an adequate degree of conversion at gingival locations of class II cavities [103,104]. Moreover, the light-curing source should be perpendicular to the curing surface of the composite to assure direct access and avoid the shadowing effect of intervening tooth structure [105]. To reduce interfacial contraction stresses, the use of lower curing rates such as soft-start curing to maintain the ability of the material to have an extended pre-gel stage to flow and deform during contraction is advocated. Lower light curing rates, however, should not interfere with attaining an adequate degree of curing. The use of low shrinkage composite is another option to reduce interfacial contraction stresses [106].

The adequate curing of resin bonding agents is essential for effective and stable bonding. The initial light curing of bonding agents is boosted by applying a layer of a flowable composite of less than 1 mm thickness at the gingival wall of class II cavities in open or closed sandwich techniques, or as an initial thin liner on the pulpal, axial, and gingival walls of class II cavities (cavity floor). Light passing through this layer facilitates further curing of the subjacent bonding agent, particularly for the air-inhibited surface layer of the resin bonding agent. Moreover, this flowable composite layer provides a zone of resilience during curing and flexibly yields during polymerization contraction of the higher stiffness composite of the subsequent increments [93,94,107]. The use of resin-modified glass ionomer liners is another alternative sandwich technique, with debates regarding the durability and degradation vulnerability [108].

The use of bulk-fill composites furnishes the advantages of reduced time and efforts of application, in addition to avoiding the incorporation of air voids during conventional incremental application with adverse influences on material properties. Bulk-fill composites have higher translucency and different photo-initiator systems to assure an adequate degree of conversion in increased thickness in comparison to conventional incremental composites. Moreover, the resin matrix of many bulk-fill composites contains contraction stress-absorbing resins to reduce the interfacial contraction stresses [103]. Flowable bulk-fill composites used as dentin replacement materials might produce a better marginal seal, particularly when the gingival margin of class II resin composites is at a deeper location gingivally [103].

Conversely, out of 140 in vitro studies and 14 in vivo investigations, a recent systematic review and meta-analysis concludes that using an intermediate layer of flowable composite cervically in the proximal box of class II resin composites does not provide an advantage in effectiveness. Upon data search and analysis, the authors favored bond strength investigations over microleakage studies. Further studies are recommended before a clear-cut conclusion can be drawn due to wide variations in the employed techniques and testing methodology of the analyzed studies [109].

Preheating the resin composite and inserting the material under sonic vibrations are reported among the suggested insertion techniques. Although these techniques improve the degree of curing, reduce internal voids, and improve strength, controversies exist regarding their validity to promote adaptation with different resin composites [110,111,112,113].

Since all resin composites show inherent limitations of unavoidable volumetric polymerization contraction, indirect restorative options might be advisable in large-size cavities so that shrinkage would take place outside of the prepared cavity. Evidence indicates that ceramic and resin composite inlays and onlays, including those for CAD/CAM technology, have excellent long-term clinical performance and can constitute better alternatives to direct composites in extensive cavities [114,115]. Immediate dentin sealing is a procedure where etch-and-rinse or self-etch adhesive resin is applied on freshly cut dentin surfaces before impression-taking in indirect restorations. The technique facilitates stress-free dentin bonds, preventing bacterial leakage and sensitivity during the temporization phase. Although the procedure improves the bonding quality and reliability of indirect restorations and elicits a better in vitro adaptation of ceramic inlays, it increases the marginal gap width of ceramic laminate veneers [116,117].

### 5.2. Biodegradation of Resin Bonds and Hybrid Layer

#### 5.2.1. Mechanisms of Biodegradation of Resin Bonds to Dentin

The oral cavity constitutes a challenging complexity that adversely affects all dental restorative materials, leading to a time-dependent gradual deterioration in restoration performance and clinical reliability. Unavoidable conditions of humidity and moisture, as well as fluctuations in functional loading, thermal and pH cycling normally occur in the oral cavity. Furthermore, natural oral habitats of microorganisms of more than 700 different species with their complicated biochemical activities, acidic and enzymatic products ordinarily exist [118]. These influencing factors act together to compete and gradually degrade dental restorations and interfacial attachment complexes [4,119]. Resin bonding to dentin is a routine practice in restorative dentistry for direct and indirect restorations [120]. The procedure involves either etch-and-rinse or self-etch approaches.

Postoperatively, gradual time-dependent hydrolytic degradation of the resin adhesives that have infiltrated the collagen plexus of the hybrid layer takes place with the leaching of resin adhesive degradational products. The process is more pronounced with poorly polymerized resin adhesives and becomes aggravated as the resin adhesive progressively degrades, exposing the previously infiltrated collagen. Water penetration and movement across the exposed collagen plexus of the hybrid layer progressively increase, creating water-filled channels and the vulnerability of denuded collagen to enzymatic proteolysis [121].

Endogenous collagenolytic enzyme MMPs and cysteine cathepsins are bound in mineralized dentin. The acidic treatment of the dentin surface activates the MMPs present in the dentin matrix in an inactive form, which becomes responsible for the degradation of collagen in the hybrid layer, together with cysteine cathepsins [122]. With the etch-and-rinse approach, the incomplete penetration of the collagen plexus leaves a denuded collagen layer at the bottom of the hybrid layer with vulnerability to the MMPs’ degradational activities [123]. Self-etch adhesive procedures involve synchronized etching and the resin infiltration of dentin collagen, avoiding incomplete resin infiltration. However, self-etch adhesives act as a water-permeable membrane, creating a water-treeing reticular fashion of water penetration leading to a characteristic nanoleakage pattern of self-etch adhesives predisposed to biodegradation [121,124].

The long-term deterioration of resin dentin interfacial bonds can be due to the degradation of the hybrid layer collagen fibrils and the hydrolytic degradation of the resin component of the hybrid layer, as well as due to endogenous host proteases and exogenous proteases produced by bacterial metabolic activities. The possible clinical adverse consequence of such deterioration includes increased hypersensitivity, recurrent caries, marginal discoloration, and the development of reversible and irreversible pulpitis [7,125]. This enzymatic degradation is further aggravated by the adverse influences of functional loading, thermal and pH cycling, and the humidity of the oral environment [126,127,128].

#### 5.2.2. MMPs Inhibitors

Four TIMPs (1, 2, 3, 4) are isolated from human tissues and fluids. These natural endogenous MMP inhibitors regulate and control MMPs’ expression and function. Each has a specific gene regulation pattern, expression profile, and binding affinity to specific MMPs [129]. Several attempts suggest inhibiting MMPs to control caries [130] and/or maintain the effectiveness of resin adhesive bonds to dentin [131,132]. One of the most widely used MMP inhibitors is chlorohexidine, which effectively and nonspecifically reduces collagen degradation via MMPs and other collagenolytic enzymes such as cysteine cathepsins. Chlorohexidine is used to control caries, to treat the dentin surface after acid etching, or is incorporated into the bonding agent to boost bonding effectiveness and longevity [130]. Different studies and systematic reviews indicate that chlorohexidine improves the long-term stability of resin bonds to dentin, with some limitations concerning the test aging periods and the need for more supportive clinical data [131].

In an experimental study, pH-sensitive nanocarriers of mesoporous silica loaded with chlorohexidine are incorporated in an experimental resin bonding agent to provide MMPs’ inhibiting effect in an acidic microenvironment produced by acid etching and dental caries [133]. The controlled release of chlorohexidine at the dentin surface by adding clays to dentin bonding agents is found to improve the durability of resin bonds to dentin [134]. Another strategy modifies resin adhesives by adding doxycycline-loaded nanotubes which inhibit MMPs without cytotoxicity or compromising the physicomechanical properties of the bonding agent [135].

Ethylene-diamine-tetra-acetic acid, tetracyclines, galardin, batmastarti, benzalkonium chloride, quaternary ammonium silane, alcohols and quaternary ammonium compounds have MMP-inhibiting potential. The application of different cross-linking agents tries to inhibit MMPs in dental caries and increase the resistance of dentin collagen degradation and improve resin dentin bond longevity. In this respect, proanthocyanidin, glutaraldehyde, riboflavin, reservaratol, and quercetin are recommended. Competing for the active sites in collagen molecules with zinc-containing compounds such as zinc oxide or zinc chloride in the resin adhesive is also proposed. Natural extracts such as carbodiimide and epigallocatechin-3-gallate are applied to the dentin surface as pretreatments before bonding procedures to induce a dual function by competing for the active sites in collagen and providing collagen cross-linking effects [136,137]. Remineralization potentials of the denuded collagen plexus using different remineralizing agents such as fluorides in fluoridated bonding agents or the incorporation of nanoparticles such as zinc oxide, silver, and copper improve the bond stability and collagen degradation resistance. Using laser treatment for dentin and modifying bonding procedures by applying a layer of hydrophobic resin are also advocated [7,138,139,140,141].

The literature indicates the effectiveness of different types of lasers for the pretreatment of dentin before applying bonding agents, as well as laser application before polymerizing bonding agents. The advantages of improved bond strength, the increased penetration of bonding agents in the superficial layer of dentin, and decreased nanoleakage have been listed [142,143,144,145].

**Figure 4:**
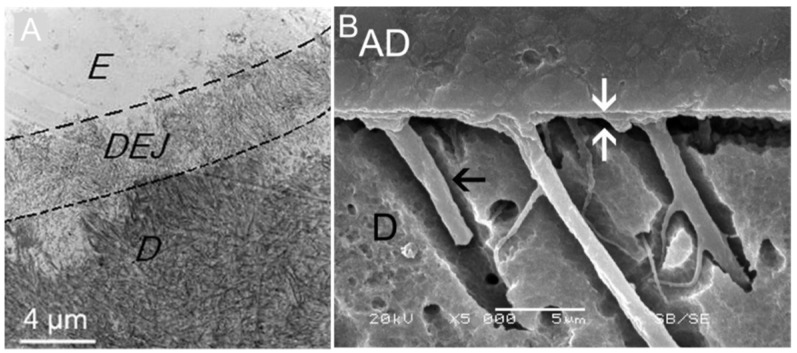
SEM microphotographic features of the dentinoenamel junction (DEJ); enamel (E); dentin (D); reused open access image from Niu et al. [146] (**A**). The resin–dentin interfaces with hybrid layers are indicated by white arrows; adhesive (AD); dentin (D); the black arrow indicates resin tags; reused open access image by Pomacóndor-Hernández et al. [147] (**B**).

An ideal resin–dentin bond should grant biomimetic junctional complexes of integration between the two structures, mimicking the dentinoenamel junction (DEJ) that integrates enamel to dentin [146,147] (Figure 4).

## 6. Contemporary Trends in Resin Composite Material Technology to Minimize the Risk of Recurrent Caries

Traditional resin composites have no antibacterial effects. Conversely, composites favor bacterial growth through eluted ethylene glycol dimethylacrylate and triethylene glycol dimethacrylate [148]. The released monomers have the potential to activate glucosyl transferase enzymes, enhancing dental plaque accumulation [149]. The biochemical interactions of resin composites in the complex oral cavity environment lead to progressive material structural and interfacial bond degradation with the eventual deterioration of marginal and interfacial adaptation. This facilitates cariogenic bacterial growth and virulence [150]. A split-mouth longitudinal clinical study indicates that caries occurs more frequently in intact proximal teeth surfaces adjacent to class II resin composite restorations than in those adjacent to intact teeth surfaces [151].

Since recurrent caries is a main form of failure of resin composites, the development of resin composite materials with bacterial biofilm-inhibiting potential is studied. The aim is to impede biofilm acidity and prevent or minimize the risk of caries recurrence, while at the same time maintaining biocompatibility and non-toxicity with satisfactory esthetic and strength properties [152]. Improvements are still needed to assure clinical applicability and long-term effectiveness without adversely influencing the esthetic and mechanical properties of the material [152,153].

A group of bioactive resin composites and bonding agents with fluoride-, calcium- and phosphate ion-releasing potential display efficient bacterial biofilm inhibition and tooth remineralization enhancement [154]. Bioactive substances can be defined as agents that would interact with tissues and living cells [155,156]. A hybrid of nanotechnology and remineralization potentials is employed; composite materials with bioactive nanoparticle-based platforms and calcium phosphate (CaP) interactions are found to be promising for halting mineral loss, inhibiting caries-related biofilms, and enhancing remineralization. Metallic nanoparticles with antibacterial activity and calcium phosphate remineralizing nanoparticles are famous examples. Amorphous-CaP, tri-CaP, tetra-CaP, di-CaP, and anhydrous and di-CaP dihydrate are tested [153]. Critical factors lead to ion release from CaP-containing composites, including pH value and CaP particle size, as greater release occurs with nanosized particles, the volume fraction of CaP, and the total filler content [153].

Attempts of imparting bioactivity to resin composites using bioactive coatings face challenges since the restoration will be subjected to the complexity of oral environmental conditions, especially for functional loading [153]. The sustained leach out of antibacterial ingredients such as silver, fluoride and chlorohexidine should be of an adequate amount. In the meantime, this antibacterial leach out should not induce cytotoxicity or undesirable deterioration in mechanical properties by leaving voids behind within the structure of the material [153,157]. To inhibit biofilm-related infections of an existing resin composite restoration, a modern concept of microbial contact killing has evolved using several bacterial contact killing agents. Retarding the level of bacterial attachment at the surface and margins of resin composites is the objective [157].

Antibacterial agents are incorporated into the surface of resin composites and resin adhesives to produce a bacterial biofilm-inhibiting effect. These agents include synthetic antibacterial compounds such as quaternary ammonium compounds, polycations and natural antibacterial agents such as antimicrobial peptides and antimicrobial enzymes. A potent long-lasting antibacterial efficiency without the development of bacterial resistance or degradation by oral bacteria, and exhibiting low immunogenicity, are basic concerns when studying these antibacterial agents. To grant bioreactivity in restorative materials, many nanoparticles are presently investigated [158].

Release-based antibacterial agents such as silver and other metallic nano-oxides such as zinc oxide and copper oxide are incorporated within the structure of the resin composite material and adhesive resin bonding agents to impart antibacterial properties by leaching out of the material at a slow rate [158]. A main concern in this technology is the adverse effect on esthetic qualities and the gradual degradation of reducing the clinical reliability of the material. Combining the contact and release of antibacterial agents are considered in dual action to help abstain undesirable consequences [157].

Using zinc oxide nanoparticles has attracted the attention of researchers because of their biocompatibility, antibacterial effects and bacterial biofilm growth-inhibiting potential. Moreover, zinc oxide facilitates bioreactivity by forming calcium phosphate on its surface. Using zinc oxide nanoparticles in resin bonding agents yielded an antibacterial effect without interfering with bonding performance and physicochemical properties [158].

On-demand antibacterial material is explored by incorporating a silver nitrate antibacterial agent into poly-isopropyl-acrylamide-co-allylamine nanogel with a capability to respond to environmental changes such as pH and temperature. However, fluctuations in pH and mouth temperature due to the intake of acidic beverages or warm drinks rather than bacterial activities might inadvertently exhaust the antibacterial effects of the material. Materials with bacterial-resistant surfaces that reduce bacterial surface attachment are investigated. In this technology, ethylene-glycol-based surfaces and zwitterion-based surfaces are the main concern [157]. These materials prohibit surface protein adsorption and reduce dental plaque biofilm.

Quaternary ammonium compound (QAC) 12-methacryloyloxydodecylpyridinium bromide (MDPB) is added to dental composites, bonding agents, and composite luting cements, together with fluorides, to provide antibacterial and remineralizing potential, in addition to the appealed bonding affinities of MDPB [141,159]. QAC dimethylaminohexadecyl methacrylate (DMAHDM) is incorporated in an experimental resin composite restorative together with barium borosilicate glass. The material successfully interferes with bacterial biofilm accumulation, thus having an encouraging potential of preventing root secondary caries in elderly and high-caries-risk individuals [160]. When DMAHDM is combined with 2-methacryloyloxyethyl phosphorylcholine or silver nanoparticles, the antibacterial potential of the composite is enhanced, and the conjugation with amorphous calcium phosphate or nanoparticles of calcium fluoride adds remineralizing potential [161].

The incorporation of MDP active monomers as micro- or nanofillers in resin composites shows the promising enhancement of resin–dentin bond stability and adhesiveness efficacy. However, incorporating MDP or DMAHDM monomers should result in the adequate monitoring of any probable adverse effects on mechanical and physical properties and any possible cytotoxicity [161,162,163].

A 10-MDP containing commercial self-etch adhesives produces a strong and stable bond to the tooth structure with the ability to chemically bond to the hydroxyapatite of the tooth, developing the characteristics of self-assembled nanolayered MDP–calcium salts at the adhesive interface. Moreover, the formation of an acid–base-resistant zone with an enhanced endurance of environmental acid–base challenges is observed. This acid–base-resistant zone of super dentin improves bond stability and resistance to the acid attacks of recurrent caries [164]. Super dentin is a reinforced acid–base-resistant zone below the hybrid layer created by bonding agents containing MDP and/or fluorides [165]. Nevertheless, the optimum infiltration and bond stability of these self-etch adhesives require selective enamel etching, scrubbing and application time for the solution to form MDP-Ca complexes [98,166].

Experimental novel resin adhesives and luting cements are produced by incorporating silver zeolite as active nanofillers with promising biofilm-inhibiting potentials. Despite encouraging clinical applicability and the potential to reduce the risk of recurrent caries with these materials, concerns exist regarding the adverse effects on mechanical properties [167,168].

Thermo-responsive polymers produce bacterial-release surfaces; poly-isopropyl-acrylamide is the most widely used polymer. Initially, the surface favors bacterial attachment. When changes in the local environment such as an increase in temperature take place, the surface attains bacterial antagonistic effects preventing new bacterial attachment and driving away the formed surface biofilm. Dual-function antibacterial surfaces that combine different antibacterial potentials of contact killing strategy with kill-and-release or surface resistance techniques are proposed to provide synergistic effects of the different antibacterial mechanisms [157].

Remineralizing the demineralized dentin is essential to potentiate bonding and prevent recurrent caries. The ion–precipitation remineralization of partially demineralized collagen in caries-affected dentin using remineralizing agents is anticipated. Remineralizing agents such as fluorides and amorphous calcium phosphates are released from resins and bioactive glass ionomers incorporating resin adhesives. The remaining inorganic mineral crystals in partially demineralized dentin act as nuclei or seed crystals of further mineralization and the growth of crystals. However, this process is not feasible when dentin collagen is completely devoid of mineral crystals in the total-etch bonding approach or at the outer layers of caries-affected dentin [119].

Modern attempts of biomimetic remineralization simulate the natural tooth structure developmental patterns to enable the remineralization of the completely demineralized collagen of dentin. Different biomimetic analogs are suggested to facilitate the biomimetic remineralization of dentin. Nanoprecursors of amorphous calcium phosphate in non-collagenous protein scaffolds, synthetic polymer-induced liquid precursors, and dentin-derived peptides are tested [169]. Bioactive synthetic peptides with positive influences and enhanced scaffold biofunctional activities and biomineralization potentials are among the suggested models [170]. Nanoprecursors of amorphous calcium phosphate stabilized by mesoporous silica nanofillers in resin adhesives are promising [119]. An experimental total-etch resin adhesive using biomimetic analogs with calcium and phosphate-releasing calcium silicate microfillers shows a promising performance to improve the long-term effectiveness of bonding to dentin and potentiate the remineralization of denuded non-infiltrated collagen at the deepest zone of hybrid layer [8].

An experimental resin composite with a three-in-one prospect of self-healing, anti-bacterial and remineralizing capabilities can be applied in resin composite and adhesive bonding agent technology. Microcapsules of the self-healing liquid of TEGDMA with N,N-dihydroxyethyl-p-toluidine as the tertiary amine accelerator and benzoyl peroxide as the self-healing initiator are added to the resin matrix. Moreover, dimethylaminododecyl methacrylate, a quaternary ammonium compound used for its antibacterial effects and nanoparticles of amorphous calcium phosphate for ion-release remineralization, is incorporated [171]. The promising aspects of this trial include the recovery of load-induced cracks without deteriorating the original mechanical properties of the material. This is coupled with the potent inhibition of bacterial plaque biofilms and lactic acid synthesis, and the acid neutralization and remineralization of initial demineralized lesions [171]. The microcapsular strategies and fabrication technology of self-healing composites including the use of polymeric capsules and silanized microcapsules of water/fluoroaluominoslicate glass have been reviewed recently. The material shows different biomechanical and esthetic limitations necessitating further improvements before in vivo investigations and clinical applicability [19,172].

A modern strategy combines mechanical reinforcement to improve strength and bioactivity by imparting antibacterial potentials of resin composites. Chitosan-integrated halloysite nanotubes in UDMA/TEGDMA-based composite presents an improvement in strength properties and enhanced bacterial biofilm-inhibiting effects [173]. Antibacterial and remineralizing potentials of a combined technology of amorphous calcium phosphate nanoparticles and dimethylaminohexadecyl methacrylate in a low-shrinkage resin composite of UDMA and triethylene glycol divinylbenzyl ether are studied. Without compromising flexure strength, this bioactive composite inhibits biofilms at restoration margins, safeguards adjacent tooth structure, and enhances restoration durability [174].

An amphipathic antimicrobial peptide coating of dentin is suggested as a two-tier system of resin composites. This material provides presumed protection by modulating the hydrophobicity of dentin, thus hindering the water diffusion-mediated degradation of resin–dentin interfacial bonds as one tier. Moreover, it furnishes an antibacterial biofilm activity, thus delaying or minimizing the risk of recurrent caries as a second tier. In vitro under clinically simulated conditions, this coating resists hydrolytic, thermal, mechanical, acidic, and enzymatic forms of degradation in addition to inducing a potent antimicrobial effect [175].

## 7. Protocols to Improve Proximal Contours and Contact Quality

The aim of restoring proximal surface contours and tight contact between adjacent teeth is to mimic the natural proximal tooth surface smoothness and convexity. The objective is to build up proximal contacts with appropriate size, shape, location and harmony with the adjoining embrasures, marginal ridge, and the adjacent contacting tooth surface. This assures effective physiological cleansing during mastication and is mandatory for maintaining favorable oral hygiene practices [19,176]. The challenge of building up tight proximal contact with direct resin composites is evident since it is a non-condensable and viscoelastic material. The challenge exists even for the packable formulations in comparison to amalgam, which is firmly condensed in class II cavities against well-contoured matrices. Nevertheless, building effective tight contact requires compensating for the polymerization shrinkage and thickness of the metallic matrix [177,178]. Evidence-based reports recommend using pre-contoured sectional matrix systems with separation rings and wedges to produce more effective tight contacts and more anatomically shaped contours with class II direct resin composites than a circumferential matrix with wooden wedges [179,180,181,182].

However, according to a cross-sectional survey in 2009, only 10% of participating dentists use the sectional matrix in class II direct resin composite restorations. In 2021, according to another survey, most dentists in the UK do not use the sectional matrix, referring to the high technique sensitivity as a reason for not using it by inexperienced dentists. Easy deformability was another listed disadvantage. The availability of different sectional matrix systems of varying materials of fabrication, opacity, rigidity, emergence profile and integrated adjoining devices means that the operator is obligated to choose the most suitable one for a particular case. This constitutes a possible additional challenge. Producing a concavity at proximal contact areas with more biofilm accumulation and difficult cleanability can occur upon using a sectional matrix, which is less likely to occur with circumferential matrices [179].

Regardless of the sectional matrix system used, the following criteria of a properly applied matrix are essential to ascertain the faithful restoration of physiological anatomical features of proximal contours, embrasures, and tight contacts [179]:

1. An appropriately adapted matrix that contacts the greatest convexity point of the adjacent tooth surface. This is influenced by the cavity design, size, and configurations as well as by the operator skills of matrix selection, placement, and stabilization method in addition to the specific resin composite used and its insertion technique.
2. Cervical seal and stability. This can be achieved using plastic/wooden wedges of appropriate size and shape, as well as separators, together with improving the cervical seal with Teflon tape or the Teflon floss method.
3. The separation of adjacent teeth to compensate for the thickness of the used matrix. This can be achieved by the employment of separators, wedges, and ring separators.
4. The coronal stability of the matrix, which is affected by matrix shape rigidity that can be secured by separating rings or by curing unbonded flowable composites against the tooth surface at peripheral matrix extensions.
5. An undistorted matrix, which is influenced by all the previously mentioned criteria and associated factors.

To assure the above-mentioned criteria, the matrix should extend cervically below the gingival margin of the preparation and about 0.5 mm above the level of the marginal ridge of the adjacent tooth [19,179]. It is worth emphasizing that rubber dam isolation is mandatory for facilitating proper bonding procedures and resin composite placement, and producing an effective tight proximal contact of resin composite restorations [19].

The extent of cavity margins, particularly gingivally, is a critical factor when deciding the most appropriate restoration, specific sectional matrix, and the insertion technique. The evaluation of interproximal spaces, clearance buccally and lingually, and the distance between the cervical margin of the cavity and the adjacent tooth surface are required before selecting a suitable matrix and stabilizing procedure. This is essential to confirm proper adaptation, the cervical and coronal seal, and the stability of the matrix [19].

In moderately deep subgingivally extending margins, saddle or perforated contoured matrices are recommended. The saddle matrix is tightened to the tooth with a special tightener and a Teflon tape wedged between the matrix band and a rubber dam is used to confirm the cervical seal and adaptation to the cervical margin of the cavity [19].

In extensive class II cavities with wide isthmus portions and cuspal involvement, an indirect restorative option in the form of resin composite or ceramic inlays or onlays is recommended for the best buildup of proximal contours and tight proximal contacts. Deep subgingivally extending margins is a clinically challenging situation that interferes with effective rubber dam isolation and the precise application of the adhesive bonding agent. Furthermore, it complicates the insertion of direct restorations and appropriate impression taking (traditional or optical) and the delivery of indirect restorations. The imprecision of the restoration’s subgingival margin leads to more biofilm accumulation, gingival and periodontal inflammation, and an increased risk of RC [11,77,78,183].

Deep marginal elevation (DME) is a technique of placing a direct composite base on a deep subgingivally located gingival wall to bring the margin to a supragingival position. The technique is an alternative to surgical crown lengthening procedures for direct and indirect restorations [184]. DME produces favorable outcomes, validating its routine use in deep subgingivally located margins [183]. When an indirect restoration is selected, immediate dentin sealing is performed with DME before impression-taking to improve restoration performance and reduce post-restorative hypersensitivity [184,185]. Special differently designed matrices are suggested during DME, including matrix in a matrix and the modified Tofflemire matrix [184,186].

An adequate degree of curing and the ability to provide a favorable seal at the critical cervical margin is crucial and should receive extra attention in DME procedures. The use of dentin for replacing flowable bulk-fill composites might be advantageous in terms of bonding effectiveness when margins are in cementum [104]. Dual-cure bulk-fill composites might provide a better degree of conversion but may not reduce marginal gaps in DME [187]. Self-etching bonding agents in deep non-enamel marginal locations are recommended rather than total-etch. Bonding agents with incorporated MDP and fluorides might be an advantageous option to impart antibacterial and MMPs’ inhibiting effects and enhance bond durability [125,188]. Partial indirect restorations with DME have good survival rates [103,189,190]. The surface roughness of DME material is critical as it might favor biofilm accumulation, particularly in deep gingival margin locations. Manual scaling was found to produce less surface deterioration than the ultrasonic scaling of DME materials, including flowable bulk-fill composites [191].

## 8. Concluding Remarks and Future Perspectives

Indistinguishable from primary caries, recurrent caries is a tooth-adherent bacterial biofilm-induced disease and an outcome of complex interactions of several factors with tooth structure. However, the pathogenicity of recurrent caries and/or the progress of residual caries is greatly influenced by the behavior and clinical performance of the existing adjoining restoration.

Presently, there is no polymerization contraction-free resin composite. Moreover, all available resin composites and established resin adhesive junctional complexes with tooth structure are prone to time-dependent degradation with the deterioration of the marginal and interfacial seals. This appears to have a strong connection with the adverse influences of oral environmental factors. The effect is more pronounced at the cervical margins of class II cavities, where the problem of recurrent caries is more frequently encountered. In clinical practice, producing a resin composite restoration with a perfect and long-lasting peripheral seal is neither predictable nor achievable.

To date, recurrent caries is a predominant cause of the failure and replacement of resin composite restorations. Although recurrent caries is initiated by local acidic production of bacterial biofilms at tooth structure sites, the exact mechanism of onset of marginal and/or wall recurrent caries is not fully elucidated. In addition to patient-related factors, the presence of gaps at the tooth–resin composite margins or interfaces seems to play a role in the development of recurrent caries. Gaps developing initially due to high polymerization contraction stresses and/or failure to achieve effective interfacial bonding, or later due to the gradual biodegradation of bonding upon restoration aging, might be controlled by using recommended evidence-based protocols. Although recurrent caries and residual caries are frequently dealt with differently, there is a lack of clear distinction between them, particularly for wall recurrent caries.

The progress of residual caries after minimally invasive incomplete caries removal in deep cavities is, therefore, a logical concern. Since it is not possible to completely prevent recurrent caries, efforts should be spent to restrain it and minimize the risk of its incidence. Careful diagnosis and analysis of existing oral environmental conditions and the extent of primary caries are fundamental. Cavity preparation has a key role in the success or failure of the restoration. The precise application of resin composite and adhesive bonding materials necessitates an ample consideration of cavity design and preparation details, including rubber dam isolation. Meticulous attention should be paid to the details of the bonding procedure, resin composite material selection, insertion technique, and light-curing criteria. The aim is to reduce interfacial contraction stresses and maximize bond efficiency, along with attaining the optimum degree of conversion, particularly for the critical and most vulnerable gingival interface of class II restorations.

The fact that there is no ideal-for-all-purpose resin composite material or application technique necessitates thoughtful individual case consideration. Extensive consideration should be given to deep subgingivally extending cervical margins. DME with suitable resin composites and bonding agents followed by direct or indirect restorative options versus surgical crown lengthening should be thoughtfully judged. Moreover, the appropriate use of the most suitable matrix system is crucial. To date, there is no accurate and precise method of assuring the arrest of residual caries or the maintenance of perfect marginal seal and gap-free interfaces. However, the non-invasive management of deep caries should be favored over complete caries excavation. To arrest residual caries in conservative deep caries management, the careful evaluation of remaining caries, judiciously deciding the end point of caries removal, and preparing peripheral walls of healthy hard dentin and sound enamel are integral aspects. This should be followed by effective bonding procedures and resin composite insertion techniques to achieve optimum peripheral seals assuring the diminishment of viable pathogenic bacteria over time. Frequent follow-ups are essential to diagnose any recurrent caries early. Promoting oral hygiene and controlling high-caries-risk status are basic inseparable parts of any restorative treatment plan.

Many recently presented resin composite restorative materials and bonding agents with various updated technologies, suggested bioactive capacities, and smart behavior are tested with encouraging results. Some bonding agents of ion release and antibacterial capabilities are already available in the market, but cannot totally prevent recurrent caries or markedly improve resin composite restoration’s long-term clinical reliability. Long-term longitudinal clinical trials should be performed to confirm the efficiency and durable clinical reliability of newly developed materials and technologies. Upcoming research should consider material performance under different patient-related factors, including oral hygiene and caries risk status. Research should continue to establish and promote precise and accurate technologies for diagnosing recurrent caries and evaluating residual caries with high sensitivity and specificity.

For the optimum inhibition of recurrent caries and the assurance of residual caries arrest, innovations should advance to develop clinically reliable smart/interactive resin composite materials and bonding agents capable of biomimetically integrating with tooth structure during function in the oral cavity. Such materials should be able to develop efficient and durable junctional complexes with tooth structure, in a way resembling the dentinoenamel and cementoenamel junctions. Nevertheless, they should exhibit competent and long-lasting biofilm-inhibiting potentials and effective tooth structure remineralizing capacity without adverse biomechanical or esthetic influences.

## Data Availability

Not applicable.
